# Recombinant expression, purification and PEGylation of Paneth cell peptide (cryptdin-2) with value added attributes against *Staphylococcus aureus*

**DOI:** 10.1038/s41598-020-69039-2

**Published:** 2020-07-22

**Authors:** Navneet Kaur, Rahul Dilawari, Amrita Kaur, Girish Sahni, Praveen Rishi

**Affiliations:** 1 Department of Microbiology, Panjab University, Chandigarh, India; 2 CSIR-Institute of Microbial Technology, Sector-39A, Chandigarh, India

**Keywords:** Antimicrobials, Molecular engineering

## Abstract

Cryptdins are disulfide-rich cationic antimicrobial peptides secreted by mouse Paneth cells and are known to exhibit potent antimicrobial activity against various deadly pathogens. Keeping in view the extremely low yield obtained from mouse Paneth cells and high cost of synthetic peptide(s), herein, we have attempted to produce cryptdin-2 in *Escherichia coli* using recombinant technology. To avoid lethal effects of peptide on the host cells, cryptdin-2 was expressed as a fusion protein with thioredoxin as fusion partner which yielded 40 mg/L protein in the soluble fraction. Subsequently, mature cryptdin-2 was cleaved from the fusion partner and purified by cation exchange chromatography. Since conjugation of poly(ethylene) glycol (PEG) has been known to improve the biological properties of biomolecules, therefore, we further attempted to prepare PEG-conjugated variant of cryptdin-2 using thiol specific PEGylation. Though the antimicrobial activity of PEGylated cryptdin-2 was compromised to some extent, but it was found to have enhanced serum stability for longer duration as compared to its un-modified forms. Also, it was found to exhibit reduced toxicity to the host cells. Further, its synergism with gentamicin suggests that PEGylated cryptdin-2 can be used with conventional antibiotics, thereby indicating its possibility to be used as an adjunct therapy.

## Introduction

Development of antimicrobial peptides (AMPs) as therapeutic agents has long been recognized as an important landmark to address the world wide challenge of microbial resistance towards conventional antibiotics. AMPs are key components of innate immune system which are produced by a broad range of organisms from prokaryotes to humans. α-defensins compose a class of AMPs that are cationic and cysteine-rich possessing a characteristic Cys1–Cys6, Cys2–Cys4, Cys3–Cys5 arrangement of their disulfide linkages^1–5^. These are constitutively expressed in intracellular granules of Paneth cells and secreted in response to pathogen exposure^6^. Paneth cell α-defensins in mice, termed as cryptdins comprise of six major isoforms-cryptdin-1 to cryptdin-6, which are processed and activated intracellularly in intestinal Paneth cells by matrix metalloproteinase-7 (MMP7) before secretion^7–9^. In contrast, human Paneth cell defensins HD5 and HD6, are processed by one or more isoforms of trypsin during or after secretion^10^. The peptides display non-specific broad-spectrum antimicrobial effect through direct membrane lysis or disruption of intracellular targets^11^. The possibility of utilizing these multifunctional molecules to effectively combat perpetually increasing incidence of antibiotic resistance among pathogens has intensified the research focusing on the development of such peptides designed with ameliorated attributes. The extraction of AMPs from natural source is an intricate process involving a large number of animals, which not only results in low yield but is also associated with contamination of indigenous molecules. On the other hand, production of synthetic peptide incurs a very high cost. In addition to cost and product quality, various inherent problems including stability, toxicity, sensitivity to proteolysis and insignificant efficacy limit the clinical application of theses peptides. Moreover, the intrinsic antimicrobial activity and relative sensitivity to proteolytic degradation may prevent expression of antimicrobial peptides in heterologous hosts such as *E. coli*^12–15^. Owing to the advancement in genetic recombinant technology, various AMPs have been expressed recombinantly in heterologous hosts with fusion partners including thioredoxin, SUMO, and baculoviral polyhedrin (Polh)^16–20^. Several attempts have been made to improve the therapeutic profiles of protein/peptide based interventions. Recent reports describe covalent modification and novel formulations of antimicrobial peptides, using polymers, lipids, liposomes, nanocapsules, nanoparticles, micelles, etc. which are biocompatible, biodegradable, and nontoxic in nature. In addition to improving metabolic as well as chemical stability, these strategies enable targeted delivery and time-controlled release of the peptides, thereby increasing the bioavailability^21^. AMP-polymer conjugates not only preserve their intrinsic antimicrobial activity, but also alleviate their toxicity and offers new functionalities^22^. Particularly, conjugation with poly(ethylene)glycol (PEG) has been extensively investigated for a range of therapeutic biomolecules to overcome the disadvantages associated with them^23,24^. There are limited reports on PEGylation of AMPs despite the fact that PEG conjugation offers important advantages such as improved solubility, reduced toxicity and decreased proteolytic susceptibility of these multifunctional molecules^25,26^.

Native (i.e. isolated from mouse Paneth cells) as well as chemically synthesized cryptdin-2 has been previously reported to possess a relatively broad spectrum antimicrobial activity against various deadly pathogens including *S. aureus*, *Y. enterocolitica* and *S.* Typhimurium^27^. Additionally, this peptide has been found to exhibit synergistic mode of action when used in combination with conventional antibiotics^28–30^. *Staphylococcus aureus* is an opportunistic pathogen causing a variety of clinical manifestations including nosocomial and community acquired infections ranging from mild skin infections to life-threatening diseases such as meningitis, osteomyelitis, endocarditis, toxic shock syndrome, bacteraemia and sepsis. Therefore, to combat such lethal infections the scientific community is exploring the possibility of using combination therapy. This is done with an aim to not only reduce the selective pressure on the pathogen but simultaneously decrease the effective concentration of the antibiotic to help eliminate the problem of antimicrobial resistance.

Therefore, in the present study, to investigate the therapeutic perspectives of cryptdin-2, we have attempted its over-expression and purification in *Escherichia coli,* utilizing fusion expression strategy based on pET-32b(+) system. Furthermore, we carried out PEGylation of the peptide after cysteine substitution mutagenesis to evaluate the effects of PEG-conjugation on the biological attributes of cryptdin-2 and its potential to be used in adjunction with the conventional antibiotics.

## Material and methods

### Reagents

Vector pET-32b(+) and host strain Rosetta-gami 2(DE3)pLysS were obtained from Novagen (Madison, WI, USA). Oligonucleotide primers used for mutagenesis were custom synthesized from Integrated DNA Technologies (IDT), USA. Methoxy-PEG maleimide reagent was purchased from JenKem Technology, USA. All the material required for Tricine-SDS-PAGE and SDS-PAGE were purchased from Bio-RAD, USA. Ni–NTA His·Bind Resins were procured from Qiagen. CM-Sepharose™ and Superdex-200™ pg matrices used for size-exclusion chromatographic processes were purchased from Pharmacia Amersham-GE, Uppsala, Sweden. All reagents used for experiments were of the highest purity grade from Sigma Aldrich, HiMedia and Fisher Scientific. *Staphylococccus aureus* (ATCC 9144) was procured from the culture collection centre of Institute of Microbial Technology, Chandigarh, India.

### Cloning, expression and purification of cryptdin-2 by fusion strategy

To improve the efficiency of expression of a disulfide-rich antimicrobial peptide, the gene encoding amino acid LRDLVCYCRTRGCKRRERMNGTCRKGHLMYTLCCR (PDB Accession No. AAB22838.1) identical to the sequence of mouse Paneth cell peptide cryptdin-2, was synthesized from GenScript (USA, Inc.) after codon optimization. The gene was cloned into NcoI-XhoI restriction sites in pET-32b(+) vector. The plasmid pET-32b(+)-Crp2 was transformed into the *E. coli* strain Rossetta-gami B (DE3)pLysS cells to obtain the over-expression of fusion protein. The heterologous expression of fusion protein was optimized by playing with parameters such as temperature, isopropyl-β-d-thiogalactopyranoside (IPTG) concentration and induction time. The expression of Trx-His-Crp2 was analyzed on 12.5% SDS-PAGE. A single transformed Rossetta-gami B (DE3)pLysS colony was used to inoculate 50 mL Luria–Bertani **(**LB) broth supplemented with 100 mg/L ampicillin and incubated overnight at 30 °C/220 rpm. 1% inoculum of overnight grown primary culture was transferred to a flask containing fresh 600 mL LB medium containing100 mg/L ampicillin and the cells were allowed to grow at 30 °C with 220 rpm shaking until the OD_600_ reached 0.6. The expression of fusion protein was induced by addition of 0.25 mM IPTG at 37 °C under shaking conditions. Cells were harvested by centrifugation at 8,000 rpm/4 °C after 4 h of incubation.

Cell pellet obtained was suspended in 20 mM Tris–Cl pH-8, 25 mM NaCl and 5 mM Imidazole and sonicated (heat system, New York) at 4 °C for 30 s sonic pulses interspersed with equal periods of rest with a total pulse time of 30 min. Sonicated samples were spun at 12,000 rpm/4 °C for 20 min. The supernatant obtained was subjected to immobilized metal affinity chromatography (IMAC) purification using Ni–NTA column (Qiagen) pre-equilibrated with 20 mM Tris–Cl (pH 8), 25 mM NaCl and 5 mM Imidazole. The column was washed with the same buffer containing 100 mM NaCl and 10 mM Imidazole and subsequently the bound protein was eluted with 500 mM Imidazole in 20 mM Tris–Cl (pH 8) and 50 mM NaCl. All chromatography steps were carried out at 4 °C. The eluted protein fractions were desalinated and renatured by dialysis against dialysis buffer (50 mM Tris–Cl, 0.5 mM EDTA, 10 mM NaCl, 10% glycerol, 1% glycine, pH 8). The amount of protein was measured using Bradford’s method^31^ and its purity was analyzed on 12.5% SDS-PAGE^32^.

### Preparation, expression and purification of single-site mutant

To generate site-specific PEG-conjugate of cryptdin-2, the residue to be targeted was selected by assessing the surface accessible epitope of the peptide using Emini Surface Accessibility Prediction database (https://tools.iedb.org/bcell/). Site-directed mutagenesis was performed to incorporate free cysteine in cryptdin-2 using QuickChange mutagenesis kit (Stratagene Inc., WI, USA). Both the strands of template were replicated with high fidelity by *pfu* turbo enzyme using a pair of complementary primers designed with the desired mutation. The non-mutated and methylated parental plasmid was digested with DpnI (Thermo Fisher Scientific, USA) and the remaining plasmids were transformed into *E. coli* XL-1 Blue competent cells, which were further validated by DNA sequencing. The mutant cryptdin-2 was expressed and purified by similar methodology that was used for wild-type cryptdin-2 fusion protein. Purified protein was dialyzed against renaturing buffer containing 50 mM Tris–Cl, 0.5 mM EDTA, 10 mM NaCl, 10% glycerol, 1% glycine, pH 8. Concentration of the purified protein was determined using Bradford reagent^31^.

### PEG-conjugation and purification of PEGylated mutant

DTNB assay using 5,5′-dithio-bis-(2-nitrobenzoic acid) was performed to ensure the presence of free thiol prior to the conjugation reaction^33^. Cysteine-directed PEGylation was performed using thiol-maleimide chemistry^23,34^, to target the lone cysteine residue previously introduced by mutagenesis at position E18C. Purified fusion protein (2 mg/mL) was added to 20-fold molar excess of PEG-malemide (5 kDa) in presence of 50 mM Tris–Cl (pH 8). The reaction was allowed to gently stir for 6 h at 25 °C under mild conditions. Crude reaction mixture was subjected to Ni–NTA chromatographic purification to remove residual PEG polymer present in the reaction. As a second step of purification, gel filtration chromatography was performed to separate PEG-conjugated protein from residual un-PEGylated forms. Briefly, crude PEGylation reaction was injected into Superdex 200 column connected to a fast protein liquid chromatography system (FPLC) and the purification was performed at a constant flow rate of 0.5 mL/min in 20 mM Tris–Cl (pH 8) at 4 °C. Elution of the protein was monitored at wavelength 280 nm.

### Cleavage of fusion tags and purification of mature peptide forms

Enterokinase cleavage was performed for the target peptides (wild-type/ PEGylated cryptdin-2), to remove the N-terminal Trx-His6 tag. The digestion reaction was performed at 22 °C for 16 h using EKMax kit (Invitrogen). For this, the purified fusion protein was first dialyzed against EKMax reaction buffer (50 mM Tris–Cl pH 8, 0.1% Tween-20 and 1 mM CaCl_2_), and then the reaction was carried out with 1 IU/mg of fusion protein. The cleavage pattern was observed on 16.6% Tris-tricine-SDS-PAGE^35^.

Further, the peptides (wild-type/ mutant/ PEGylated cryptdin-2) were purified from the cleaved Trx tag and undigested protein by cation-exchange chromatography. Briefly, 0.1% acetic acid was added to the reaction mixture and loaded onto CM-Sepharose column pre-equilibrated with 20 mM sodium phosphate (pH 7.2) buffer. UV absorbance at 215 nm was monitored and the peptide fractions were finally eluted with gradient of 1 M NaCl in 20 mM sodium phosphate (pH 7.2) Peptide concentration was determined on the basis of molar extinction coefficient of chromophoric residues at 215 nm. The purified peptide fractions (wild-type and PEGylated cryptdin-2) were checked for their purity on 16% Tricine-SDS-PAGE. Mass values of the purified peptides were determined by MALDI-TOF (matrix-assisted laser desorption ionization time of flight mass spectrometry) on ABISCIEX, TripleTOF 5,600/5,600 machine.

### In vitro assessment of antimicrobial activity

#### Radial diffusion assay

Antibacterial activity of all the cryptdin preparations was evaluated by performing radial diffusion assay^36^. In brief, bacteria grown in LB broth to mid-logarithmic-phase (10^6^–10^7^ CFU) were added to an overlay solution (10 mL LB with 1% agar) and poured uniformly over a petri dish containing 25 mL of 3% agar in LB broth. Following the addition of 20 μL aliquots of the cryptdin-2 preparations along with control sample (PBS) into a series of wells made in the gel, the plates were incubated overnight at 37 °C. The samples were examined for zone of clearance around the wells indicating inhibition of bacterial growth.

#### Determination of minimum inhibitory concentration (MIC)

To determine MIC values of the peptide preparations, micro broth dilution assay was performed^37^. Briefly, *S. aureus* cells were grown to mid-exponential phase and diluted to a concentration of approximately 10^7^ CFU/mL. Different concentrations of AMP preparations were separately added to wells containing bacterial suspension (approximately 10^6^ CFU/mL) in sterile 96-well flat-bottom plate. After incubation at 37 °C, cell growth inhibition was assessed by measuring the absorbance at 595 nm on microplate reader (BioTek). MIC was recorded as the lowest concentration of the peptide preparation at which no visible growth was observed.

#### Flow cytometry assay

Flow cytometry (FCM) studies were performed to evaluate bacterial cell membrane integrity of peptide treated cells^38^. In brief, *S. aureus* cells grown in LB to mid-exponential phase were washed with PBS. The bacterial suspensions containing 10^7^ CFU/mL were incubated with the peptide preparations (MIC) for 1 h at 37 °C. The treated cells were harvested, washed, and then incubated with 10 µg/mL 7-Aminoactinomycin D (7‐AAD) dye at room temperature. After 15 min of incubation, the unbound dye was removed by washing with PBS. The data was acquired with an excitation wavelength of 488 nm on FCM instrument (BD FACSVerse™|Flow Cytometer). Bacterial cells incubated with 7‐AAD in the absence of any peptide preparation were used as the negative control.

#### Scanning electron microscopy

The surface morphology of *S. aureus* treated with wild-type cryptdin-2 and its PEGylated variant was studied by Scanning Electron Microscopy (SEM) analysis^39^. Briefly, *S. aureus* cells were grown to mid-logarithmic phase, washed and resuspended in PBS. The recombinant peptide variants (2 × MIC) were added separately to the tubes containing 10^8^ cells/mL and incubated for 1 h at 37 °C. Post treatment, cells were washed with PBS and fixed with Karnowsky´s fixative. Subsequently, the samples were dehydrated using ethanol and were imaged by Scanning Electron Microscope (Zeiss EVO40).

#### Cell cytotoxicity assay

The cytotoxicity of recombinant peptide variants towards RAW 264.7 cell lines was assessed using AlamarBlue (Resazurin), a cell metabolic activity reagent (Sigma)^40^. The log phase cells diluted (2 × 10^5^/well) in Dulbecco's Modified Eagle Medium (DMEM) containing 10% fetal bovine serum (FBS) were cultured in 96-well microtiter plates and incubated for 12 h under 5% CO_2_ conditions at 37 °C. Subsequently, media was removed and cells were incubated with each peptide (at their respective MIC values) at 37 °C for 24 h. Following this, 0.02% Alamar blue reagent was added to the cells and incubated for 8 h. Fluorescence was measured with excitation wavelength at 545 nm and emission wavelength at 590 nm. The percent difference between treated and un-treated cells was calculated to determine percent cytotoxicity.

#### Serum stability

The stability of purified peptide preparations in mice sera was evaluated as described previously^15^ with some modifications. Briefly, 80 µL of 50% serum was added to each well of 96-well plates followed by addition of 10 µL of bacterial suspension. The total CFU/mL value was adjusted to be identical in all the samples. Each peptide preparation (10 μL) was added to different wells and incubated at 37 °C for 0, 3, and 6 h respectively. The antimicrobial activities were assessed by performing micro broth dilution assay as described above for determination of MIC against *S. aureus*. The sera of mice were confirmed for absence of any intrinsic antimicrobial activity prior to performing the test.

#### Fractional inhibitory concentration (FIC) determination

To investigate the interactive effect of these recombinant peptides, a checkerboard assay^41^ was performed using a clinically prescribed antibiotic (gentamicin) as the other antibacterial agent. The assay employs the same methodology followed for determination of the MIC as described above. MIC values of peptide and gentamicin were used in this assay in order to calculate the FIC values. All the three peptide variants and gentamicin were tried in different ratios to evaluate interaction of the agents in combination against *S. aureus.* The same was determined on the basis of the FIC index, wherein an index of ≤ 0.5 indicated a synergistic effect and an index > 0.5 and ≤ 2 indicated an additive or indifferent nature of interaction respectively and lastly, the interaction was confirmed to be antagonistic when the index was > 2.0.

FIC index is expressed as:

$${\text{FIC Index }} = {\text{ A}}_{{1}} /{\text{A }} + {\text{ B}}_{{1}} /{\text{B}}$$

where A_1_ and B_1_ are the respective concentrations of gentamicin and peptide producing the combined MICs, while A and B are the MICs of the individual agents.

## Results

### Cloning, expression and purification of cryptdin-2 and its mutant by fusion strategy

**Figure 1:**
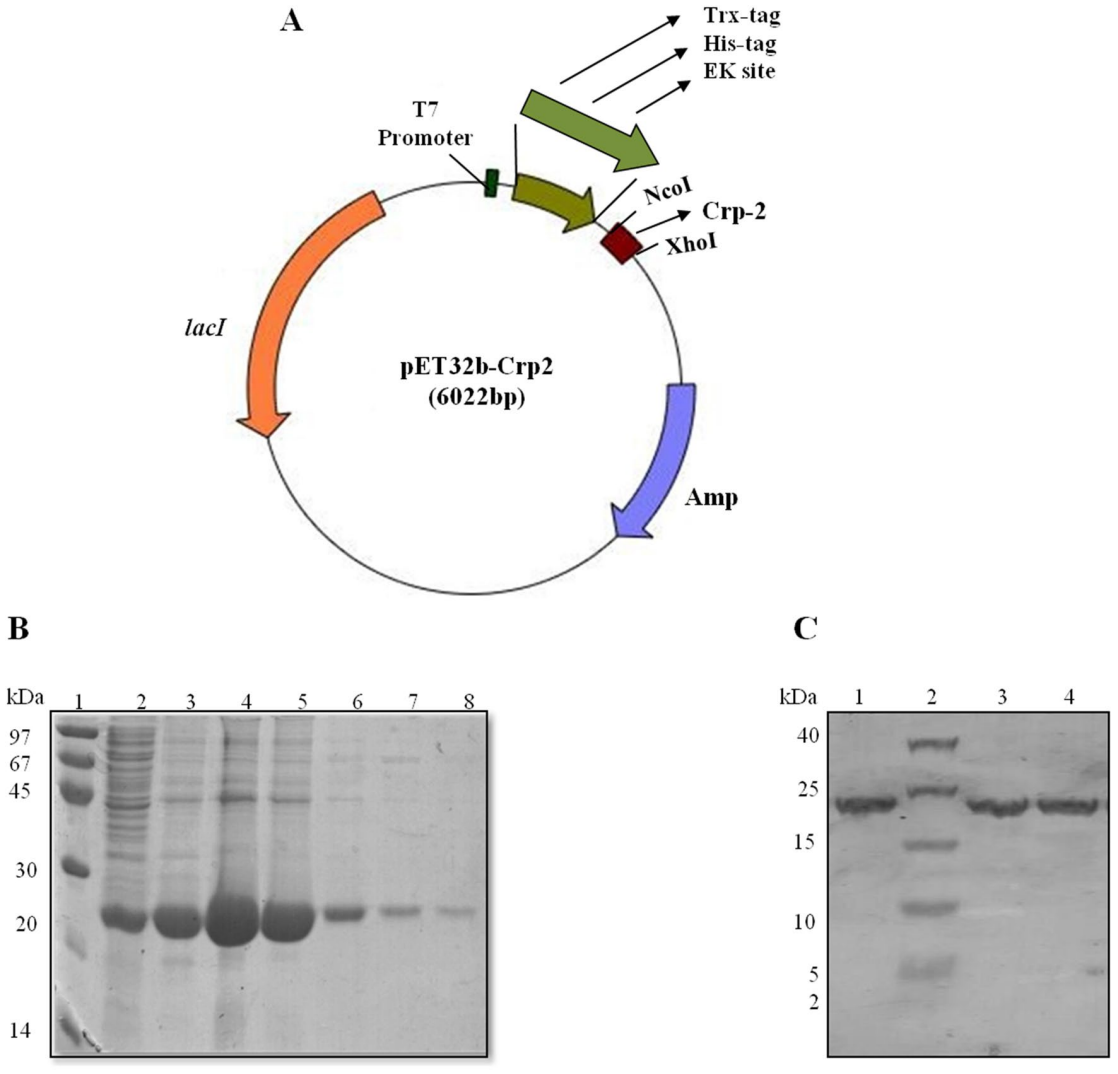
Expression and purification of cryptdin-2 by fusion strategy. (**A**) Diagrammatic representation of expression vector cassette containing thioredoxin (Trx) and histidine (His6) sequence followed by an enterokinase cleavage site (DDDDK) upstream the target peptide to be expressed. (**B**) 12.5% SDS-PAGE profile showing Ni–NTA purified fractions (Lane 3 to 8) of cryptdin-2 fusion protein eluted with 500 mM Imidazole. Lane 1. MWM; Lane 2. Preload (soluble fraction); Lane 3–8. Purified protein fractions (**C**) 16.6% Tricine gel profile displaying purified cryptdin-2 fusion protein and its thiol mutant. Lane 1. cryptdin-2 fusion protein; Lane 2. Low-range molecular weight marker (LMWM); Lane 3 and 4. Purified fusion protein mutant.

The synthesized gene encoding disulfide-rich mouse Paneth cell cryptdin-2, was cloned between NcoI-XhoI restriction sites in pET-32b(+) vector as shown in Fig. 1. The recombinant plasmid harboring cryptdin-2 gene fused with N-terminal tag was transformed into *E. coli* and cryptdin-2 fusion protein was found to be non-toxic to the host bacterium *E. coli*. The cells induced with 0.25 mM IPTG were found to express major fraction of protein in the soluble form rather than in sonicated precipitates. Subsequently, the purification of fusion protein was achieved by a single step Ni–NTA chromatography as evidenced by appearance of a clean sharp band of fusion protein when run on SDS-PAGE. The additive molecular weights of cryptdin-2 (~ 4.5 kDa) and the co-expressed fusion tags (His-tag, Trx-tag and S-tag) (17.42 kDa) corresponded to the band of fusion protein exhibiting a net molecular weight of ~ 22 kDa. The average yield of purified fusion protein after dialysis was calculated to be 40 mg/L.

Subsequently, the central region composed of KRRERM residues was predicted to possess highest accessibility and served as the most appropriate target to generate site-specific PEG-conjugate of cryptdin-2. Further, to get down to a single residue, a well-documented strategy that involved decreasing the anionic character of peptide to augment its antimicrobial potential by increasing the electrostatic interactions driven by their positive charge with the negative charged cell membranes of the microbes was followed^42–45^. Therefore, glutamic acid at 18th position lying in the surface accessible region was chosen for selective modification. Substitution of glutamic acid with cysteine (E18C) was successfully performed by following standard protocols for site-directed mutagenesis using pET-32b plasmid construct carrying wild-type cryptdin-2. The native cysteines present in the peptide sequence ultimately forming intra-molecular disulfide bonds, were left undisturbed with the purpose of maintaining the native structure as well as the functionality of the peptide. The mutant (E18C) was successfully expressed in soluble fraction and purified by Ni–NTA chromatography using similar methodological approach followed for wild-type cryptdin-2, with slight modifications. The yield of E18C cryptdin-2 fusion protein was 25 mg per litre as calculated by Bradford assay.

### PEG-conjugation and purification of PEGylated mutant

**Figure 2:**
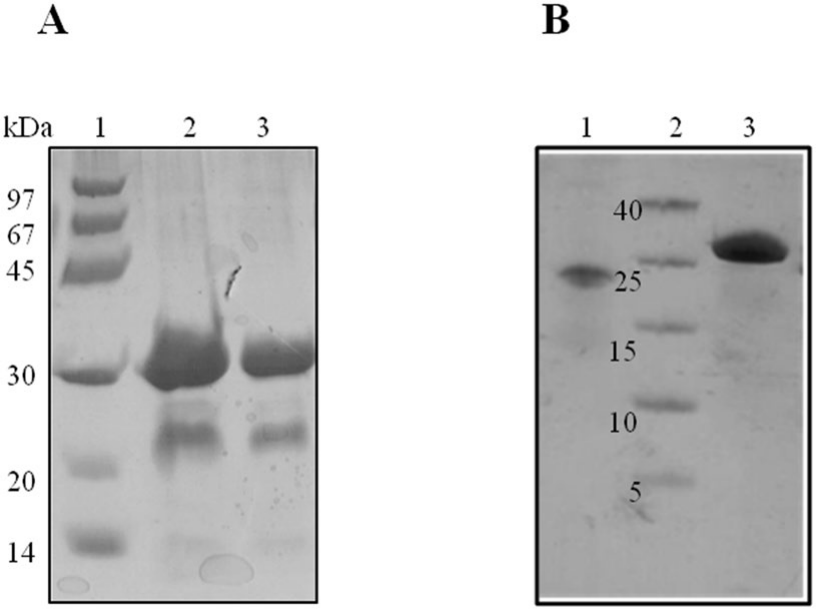
PEGylation and purification of PEG-conjugate. (**A**) SDS-PAGE profile of PEG-conjugation showing Lane 1. Molecular weight marker (MWM); Lane 2. Crude PEGylation mix; Lane 3. Ni–NTA purified PEGylation mix. (**B**) SDS-PAGE profile displaying Lane 1. Un-PEGylated cryptdin-2 fusion protein; 2. MWM; 3. Purified PEG-conjugated fusion protein.

The results of thiol group determination with DTNB assay in purified preparations of wild-type as well as the mutant peptide established the presence of single free thiol group that was incorporated by substitution mutagenesis. Thereafter, E18C mutant of cryptdin-2 fusion protein was conjugated to non-toxic FDA-approved PEG polymer having an average molecular weight of 5,000 Da. To preserve the native-like disulfide linkages in the protein, PEG-conjugation reaction was carried out under non-reducing conditions. Subsequently, un-reacted PEG fraction was removed from crude reaction mixture by Ni–NTA as first step chromatography (see Fig. 2). Further, separation of desired mono-PEGylated population of protein from residual un-reacted protein was achieved by gel-filtration chromatography. PEG-conjugates displayed slightly retarded mobility on gel which could be attributed to increased hydrodynamic volume as a result of PEG coupling^46^.

### Release and purification of cryptdin-2 and its PEGylated variant from fusion tag

**Figure 3:**
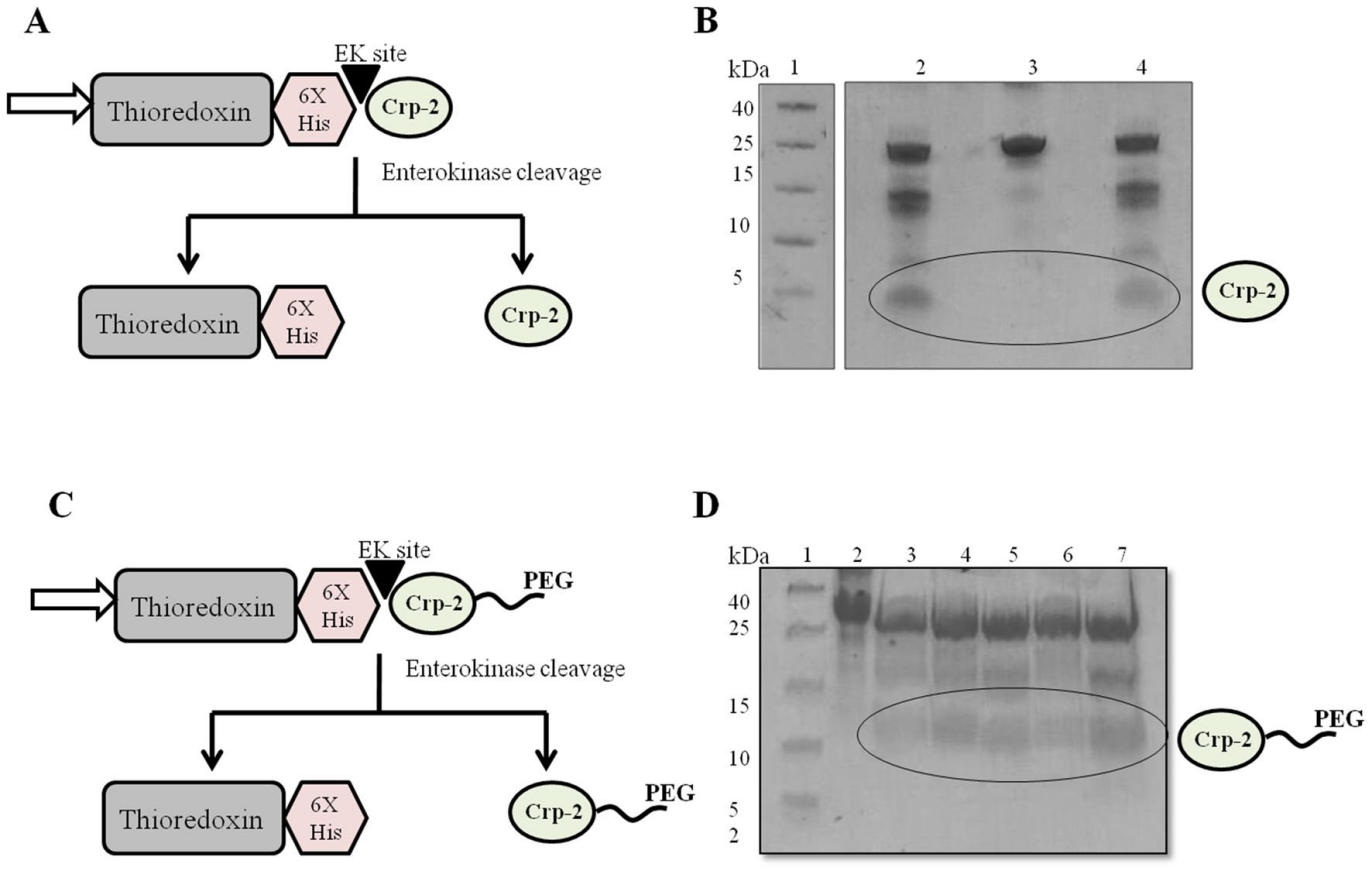
Schematic illustration of enterokinase-mediated cleavage. (**A**,**B**) Diagrammatic representation as well as SDS-PAGE pattern of EK-mediated cleavage to release cryptdin-2 and N-terminal fusion tag (Trx-His-Crp2). Lane 1. LMWM (molecular weight marker was run on a separate gel and the size of uncleaved Trx-His-Crp2 already been validated in Fig. 2B); Lane 2 and 4. EK cleavage reaction for 16 h at 22 °C; Lane 3. Cryptdin-2 fusion protein. (**C**,**D**) Diagrammatic representation as well as SDS-PAGE pattern of EK-mediated cleavage to release PEGylated cryptdin-2 and N-terminal fusion tag (Trx-His-Crp2). Lane 1. LMWM; 2. PEG-conjugated fusion protein; Lane 3 and 6. EK cleavage reaction for 8 h and 16 h at 4 °C respectively; Lane 4 and 5. EK cleavage reaction for 8 h and 16 h at 22 °C respectively; Lane 7. EK cleavage reaction for 18 h at 22 °C.

**Figure 4:**
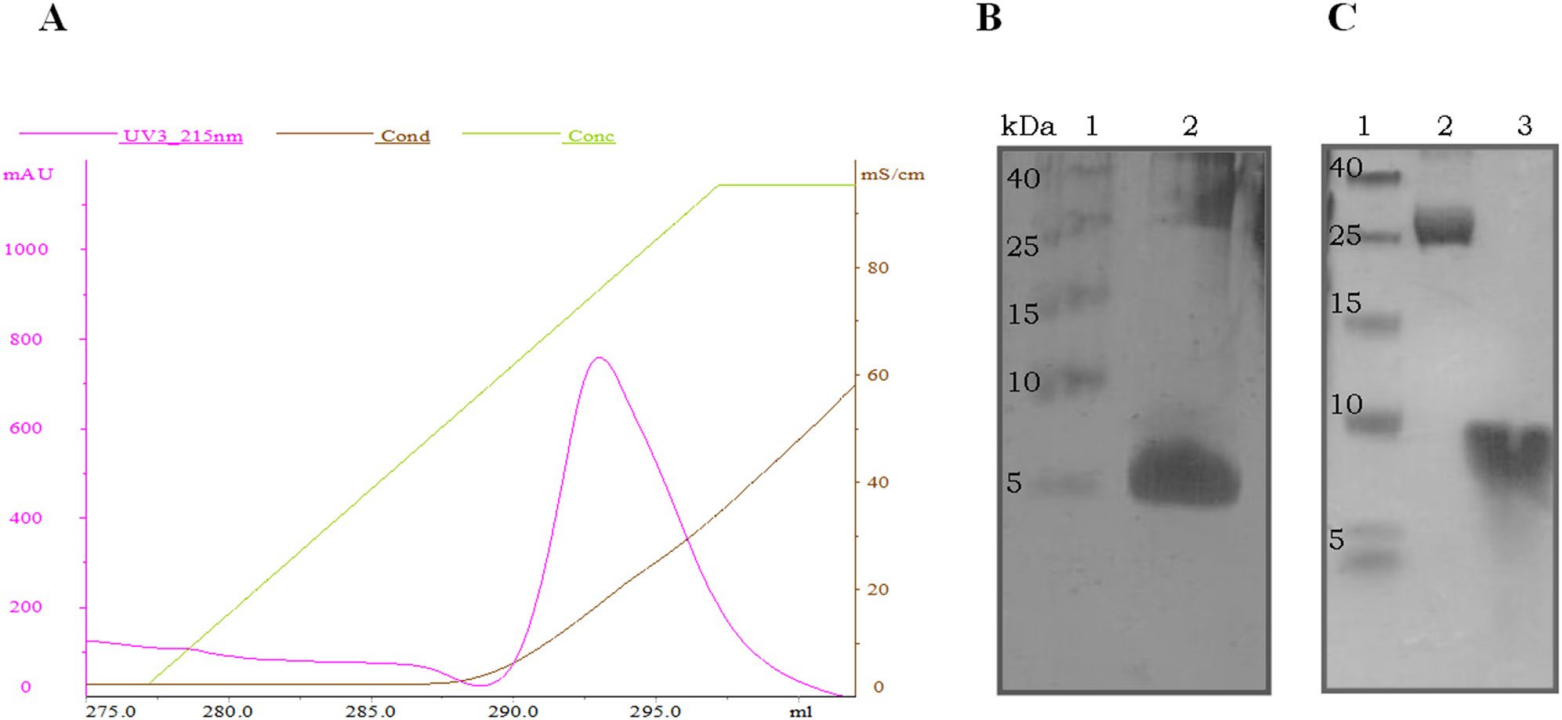
Purification of cryptdin-2 and its PEGylated variant from Trx fusion tag. (**A**) CM-sepharose chromatographic profile showing elution peak of cryptdin-2 with NaCl gradient. Similar profile was obtained in case of PEGylated variant of cryptdin-2. (**B**) Tricine SDS-PAGE showing band corresponding to purified cryptdin-2 peptide. Lane 1. LMWM, Lane 2. cryptdin-2. (**C**) Tricine SDS-PAGE of purified PEG-conjugated cryptdin-2. Lane 1. LMWM, Lane 2. PEGylated cryptdin-2 fusion protein. Lane 3. PEGylated cryptdin-2.

**Figure 5:**
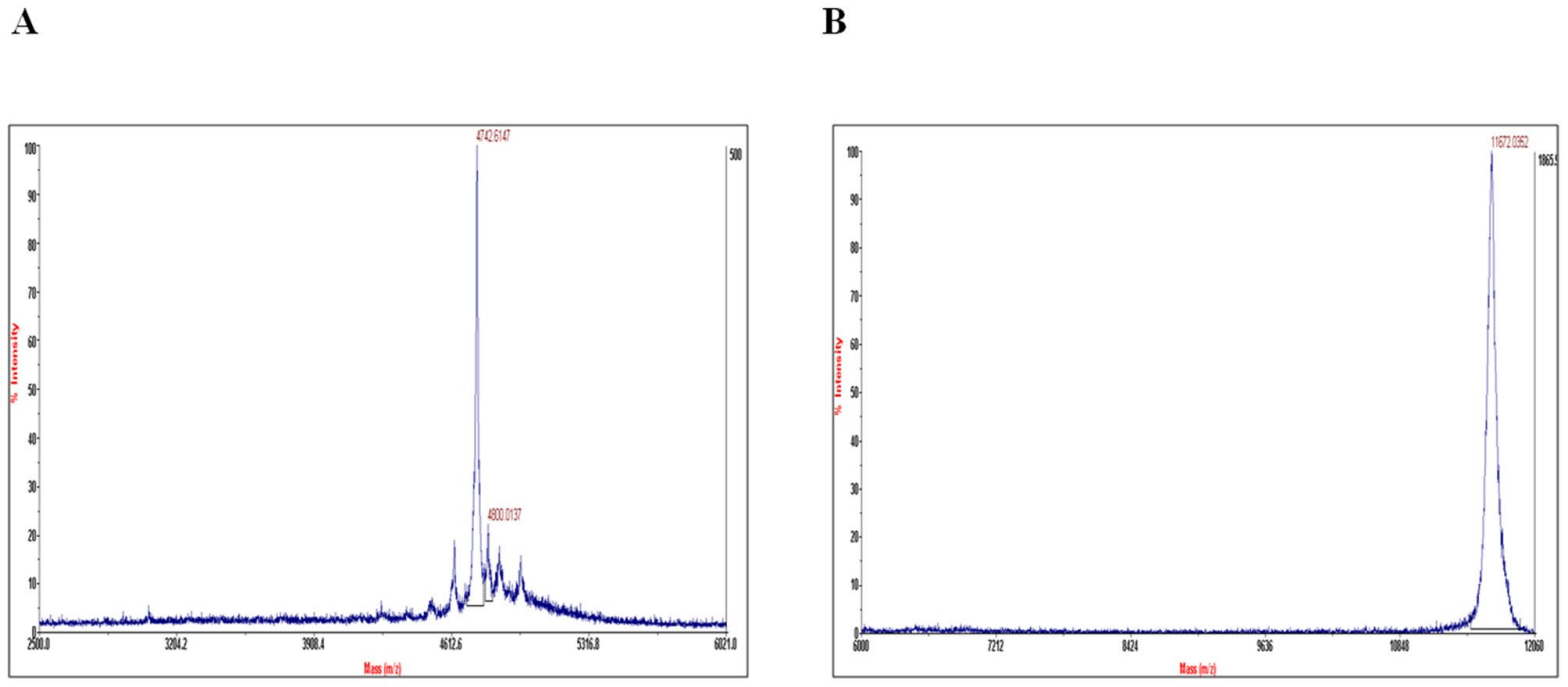
Mass spectrometric analysis of purified recombinant peptides. (**A**) MALDI-TOF peak corresponding to purified cryptdin-2 (4.742 kDa) depicts size close to the theoretical size of cryptdin-2 (~ 4.324 kDa). (**B**) MALDI-TOF peak of purified PEGylated cryptdin-2 (~ 10 kDa) which is quite close to the expected size of cryptdin-2 PEG conjugate representing an additive molecular weight.

**Figure 6:**
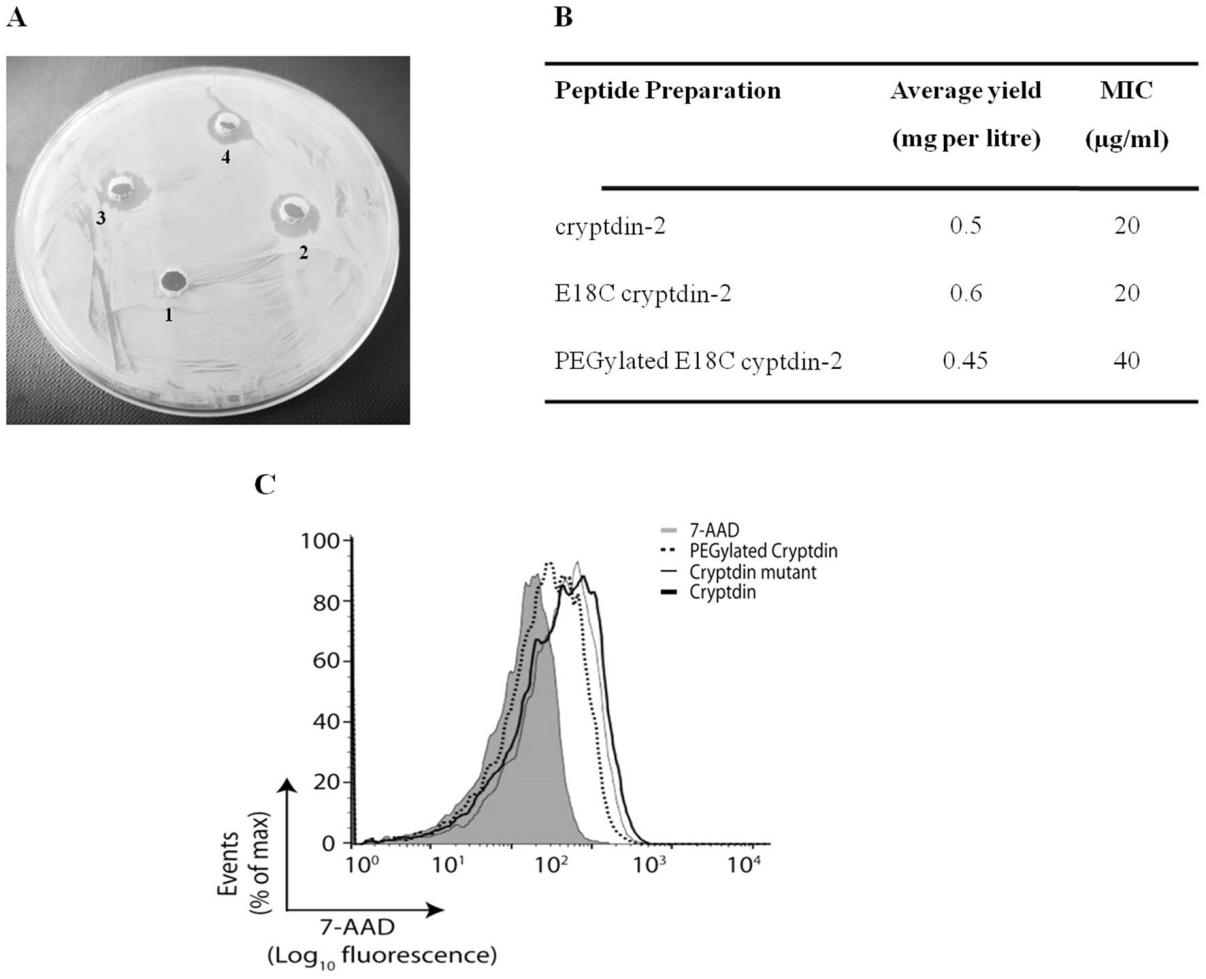
Antimicrobial activities of recombinant peptides against *S. aureus*. (**A**) Radial diffusion assay with peptide concentration 100 µg/well 1. PBS (control); 2. cryptdin-2; 3. E18C cryptdin-2; 4. cryptdin-2 PEG-conjugate. (**B**) Table displaying average purification yield and MIC values of purified peptides. (**C**) The data acquired from flow cytometry represents the dead cell population of *S. aureus,* upon treatment with each of the three peptide preparations for 1 h with each of the three peptide preparations. A histogram overlay of all the samples including control was obtained by FlowJo v10.0.7 software.

Mature target peptide was generated by cleavage of N-terminal Trx-His6 tag removed by using enterokinase. The scheme of fusion tag cleavage along with the SDS-PAGE pattern of EK digestion reaction has been depicted in Fig. 3. Purity of the respective peptides separated from the cleaved tag and undigested fraction was confirmed by Tris-tricine SDS-PAGE profile (Fig. 4). The molecular weights of both the peptides (cryptdin-2 and PEGylated cryptdin-2) were confirmed by mass spectrometry (Fig. 5). The average peptide yield calculated on the basis of molar extinction coefficient of chromophoric residues is depicted in Fig. 6B.

### Antimicrobial activity of peptides against *Staphylococcus aureus*

#### Radial diffusion activity

Agar well diffusion assay indicated that cryptdin-2, E18C-cryptdin-2, and PEG-conjugated cryptdin-2 possessed potent antimicrobial activity against *Staphylococcus aureus*, which was evidenced by the zone of growth inhibition (Fig. 6A). Although, we could not estimate the difference in activities, the results provided us with a preliminary confirmation for the presence of antimicrobial activity in all the three peptide preparations.

#### Minimum inhibitory concentration (MIC)

The MIC results revealed that PEGylated cryptdin-2 possessed lesser antimicrobial effect against *Staphylococcus aureus* in comparison to its un-PEGylated analogs as depicted by twofold decrease in its activity. MICs for cryptdin-2, its mutant and PEGylated analogs are depicted in Fig. 6B.

#### FACS analysis to determine membrane integrity

The shift in cell population was observed in gated area after 7-aminoactinomycin D (7-AAD) staining post treatment with cryptdin-2 variants. It was found that cryptdin-2 shifted the population to maximum signal suggesting maximum killing followed by mutant and its PEGylated form (see Fig. 6D). The data obtained was plotted in a histogram, which depicted maximum killing i.e. approximately 33% in wild-type cryptdin-2 treated cells. However, the relative percentage slightly declined to 25% in bacterial cells treated with PEGylated cryptdin-2.

#### Scanning electron microscopy analysis

**Figure 7:**
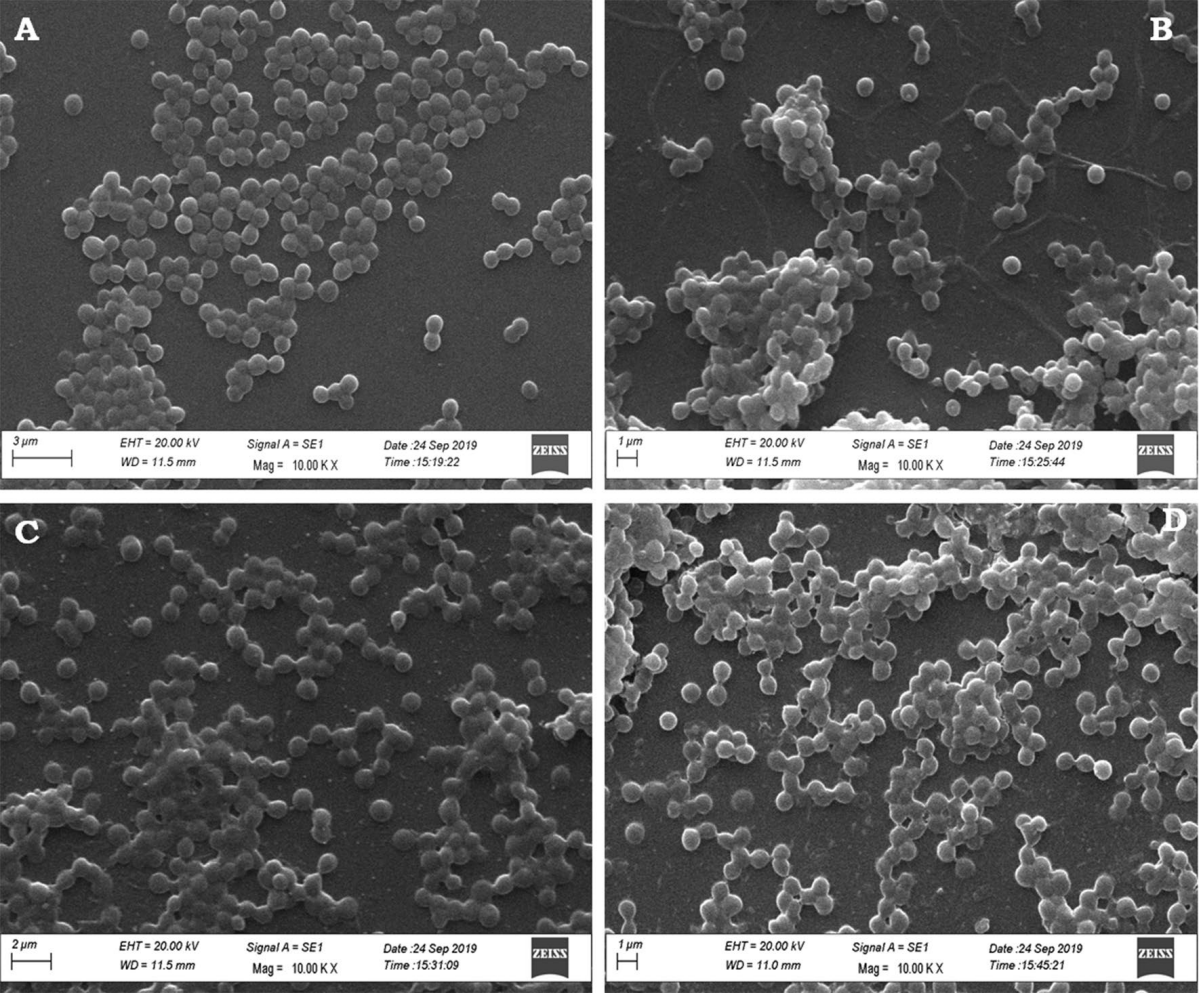
SEM micrographs of *S. aureus* showing effect of peptide treatment (2 × MIC). (**A**) Untreated *S. aureus* cells showing normal morphological characteristics. (**B**) *S. aureus* cells treated with cryptdin-2 showing abnormal cell aggregations, along with surface alterations. (**C,D**) Abnormal effects on cell surface appearance were observed in *S. aureus* cells treated with cryptdin-2 mutant (**C**) and PEGylated cryptdin-2 (**D**).

SEM images of cells treated with cryptdin-2 variants were compared to that of un-treated cells to analyze the morphological changes in *S. aureus*. Un-treated *S. aureus* cells displayed normal coccal morphology with intact and smooth surface (Fig. 7A). In contrast, treated *S. aureus* cells demonstrated altered cell surface appearance in all the three cases including wild-type crypdin-2, its mutant and the PEGylated variant (Fig. 7B–D).

#### In vitro cell cytotoxicity and serum stability

**Figure 8:**
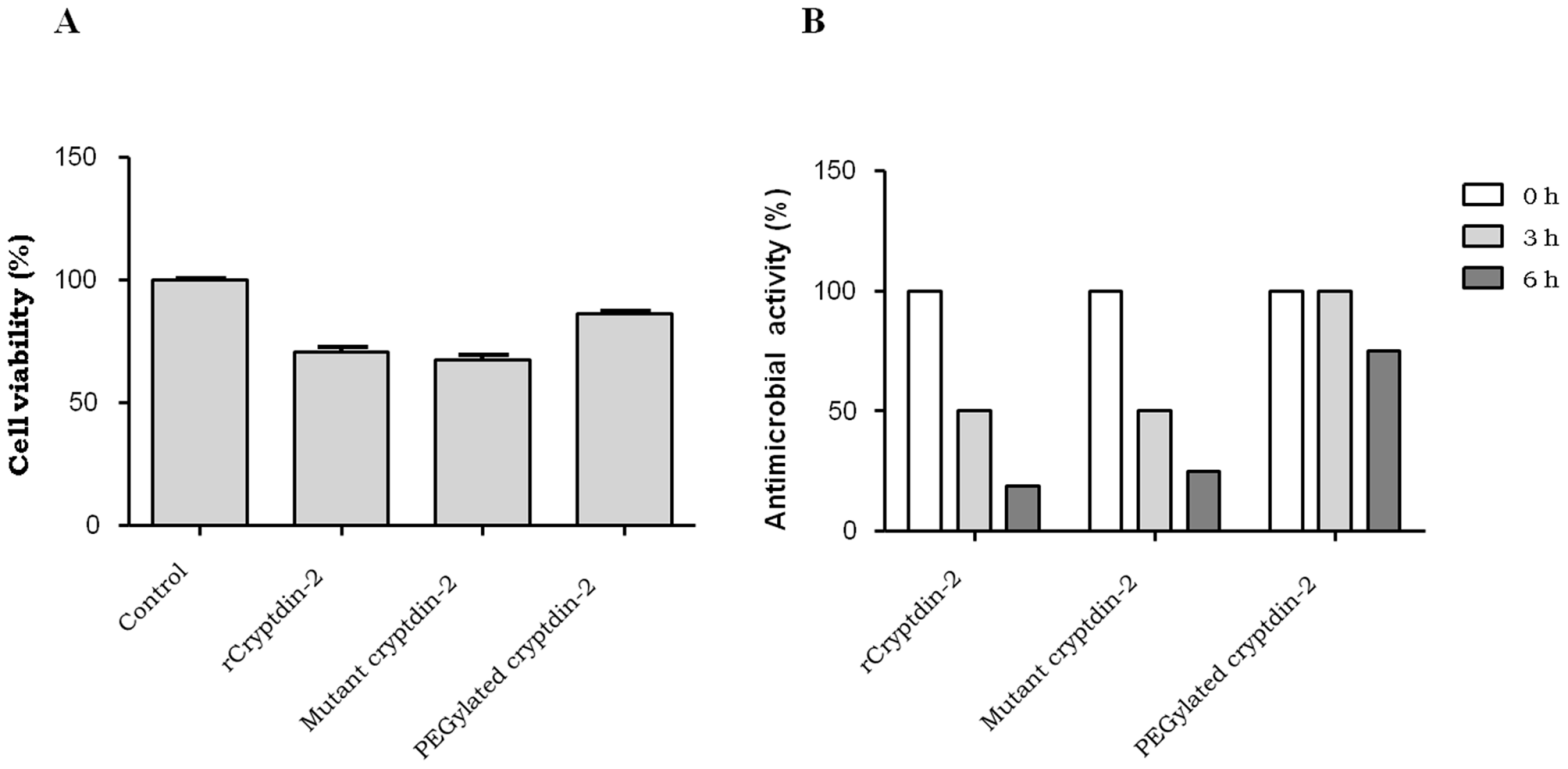
(**A**) Cell viability percentage of RAW macrophages treated with cryptdin-2 peptide preparations. The cytotoxic effects of PEG modified cryptdin-2 on murine macrophages were evaluated and compared with those of un-conjugated cryptdin-2 preparations. PEG-conjugated cryptdin-2 showed reduced toxicity towards host cells. (**B**) Percentage of antimicrobial activity in presence of sera. The stability of PEGylated and un-PEGylated cryptdin-2 analogs was tested in presence of fresh mice sera. *These experiments were repeated thrice in triplicates and the graphs shown here are indicative of the respective individual experiment.

The data obtained by performing Alamar blue test was plotted as bar graph depicting the percent difference between treated and un-treated cells to evaluate the level of toxicity. The results revealed that post treatment with peptides at their respective MIC(s), RAW macrophages were significantly viable showing approximately 70% and 65% cell viability percentages in case of cryptdin-2 and its mutant, respectively. However, the cell viability increased to approximately 87% in response to PEG-conjugated cryptdin-2 (Fig. 8). 1 × phosphate-buffered saline with Tween^®^ detergent (PBST) was used as negative control and gentamicin was used as positive control.

Additionally, incubation of PEG-conjugated cryptdin-2 at its MIC with mice sera demonstrated no change in antibacterial activity up to 3 h and approximately 30% reduction in activity up to 6 h post incubation whereas wild-type cryptdin-2 and its mutant lost 50% of its antimicrobial activity after 3 h of incubation with sera thereby indicating higher serum stability of the PEGylated peptide.

#### Fraction inhibitory concentration (FIC)

The interaction of recombinant cryptdin-2 variants in combination with clinical antibiotic (gentamicin) was studied and compared to their individual antimicrobial potency against *S. aureus.* The results revealed that MICs of recombinant peptide analogs together with gentamicin was reduced by eightfold, manifesting a more pronounced antimicrobial effect (Table 1). Equivalent values of FIC indices i.e. 0.375 were observed, in case of cryptdin-2, mutant as well as PEGylated peptide when used as an adjunct with gentamicin indicating no change in antimicrobial profile of the recombinant peptides.

**Table 1:** MIC values and FIC indices of cryptidin-2 preparations alone and in combination, against *Staphylococcus aureus*.

Antimicrobial agent	MIC (µg/mL)	MIC (µg/mL) in combination		FIC		FIC Index
		Peptides	Gentamicin	Peptides	Gentamicin	
Gentamicin	0.097					
Cryptdin-2	20	2.5	0.024	0.125	0.25	0.375
Mutant (E18C)	20	2.5	0.024	0.125	0.25	0.375
PEG-conjugated	40	5	0.024	0.125	0.25	0.375

## Discussion

A multitude of preliminary investigations indicate that cryptdin-2 is a propitious candidate for developing efficient antimicrobial therapies. Previous studies from our lab have shown that cryptdin-2 exhibits significant antimicrobial activity against various pathogenic microbes^27–29^, which is mainly attributed to its relatively strong electrostatic interaction with negatively charged bacterial membranes, eventually resulting in cell lysis. Besides their beneficial antimicrobial effects, such small cationic antimicrobial peptides have been observed to possess numerous pragmatic issues such as plausible toxicity to eukaryotic cells, low stability and rapid clearance from kidney, all of which are a matter of concern for their translational credibility^47–49^. Furthermore, limited availability in vivo and high cost of production through synthetic route hampers the widespread usage of such multifunctional peptides^50–53^. More so, the production of cryptdins is challenging due to the presence of three intramolecular disulfide linkages in such a short peptide sequence. To address these issues, we have exploited a recombinant system that allows heterogeneous expression for better yield of the desired protein/peptide. Considering the fatal toxicity of cryptdins towards *E. coli* expression host as a major bottle-neck for its recombinant expression^54^, it has been co-expressed with thioredoxin which is known to neutralize the toxic effects of AMPs towards the host cells, simultaneously acting as a solubility enhancer^55–57^. Using pET Trx Fusion System, soluble expression of the desired fusion protein in double mutant *trxB*^*−*^*/gor*^*−*^ strain of *E.coli* was successfully achieved. The coordinated performance of thioredoxin (pET-32b) and Rossetta-gami (DE3) cells (*trxB* and *gor* mutations) maintains an oxidizing milieu, facilitating disulfide bond formation in the cytoplasm, hence maximizing the level of soluble, active, and properly folded target protein. These results are similar to those reported in another study establishing the critical importance of correct disulfide bond pattern for bioactivity of HD-5 (human analogue of cryptdins)^58^, and it may be reasonable to make a similar inference from the high bio-activity obtained after the in vitro folding results that were observed. Nevertheless, it may be worth mentioning that the presence of only a single fold remains unambiguous in the light of the study by Wu et al. showing the same antibacterial activity in seven differently folded, oxidised hBD-3 preparations demonstrating the caveats involved^59^.

Further, to elucidate the functional consequences of surface modification, we prepared a site specific mutant of cryptdin-2. Taking advantage of absence of free non-native cysteine in cryptdin-2, we attempted to genetically incorporate an unpaired cysteine residue in the peptide sequence to facilitate site specific cysteine labeling for thiol mediated PEGylation. Therefore, glutamic acid (E18) lying in a region having better solvent accessibility was chosen and mutated to cysteine, which was expected to additionally enhance the antimicrobial activity by increasing the net positive charge of the peptide^60–63^. The presence of free thiol group (as validated by thiol determination assay) and an equivalent antimicrobial activity exhibited by mutant analogue indicated that cysteine substitution certainly did not affect the primary activity in terms of minimum inhibitory concentration and apparently confirmed an intact disulfide arrangement.

Following this, a comparative analysis of inhibitory activity of PEG-conjugated cryptdin-2 with its un-conjugated counterparts revealed that PEGylation had affected the MIC of the peptide. The results obtained from FACS analysis revealed compromised cell membrane integrity on treatment with wild-type, mutant and PEGylated cryptdin-2. However, lesser adverse effect was observed on cell membrane when treated with the PEGylated peptide. These findings correlated well with the increased MIC of the peptide as observed above. It is highly probable that site-specific mutation followed by PEG-conjugation influenced the antimicrobial effectiveness of the peptide. This could be attributed to the charge-masking effect of PEG moiety, thereby resulting in diminished activity. Evidences supporting these results have shown that PEGylation affects the antimicrobial activity of nisin^25^, tachyplesin I^64^, and magainin-2^65^. Additionally, it may also be mentioned that the extent of reduction in antimicrobial activity likely varies depending upon the peptide. As reported by Imura and his group, the antimicrobial activity decreased by fourfold in case of magainin 2 after conjugating it with 5 kDa PEG at N-terminal end^65^. However, the decrease in activity was more prominent i.e. 32–64-fold, in case of tachyplesin I conjugated with a PEG moiety of the same size (5 kDa) at the same position^64^. Surprisingly, the PEGylation of nisin via amine group of the lysine side-chain led to an inactive conjugate as its entire original activity was lost^25^. The delicate equilibrium of lipophlicity/hydrophilicity in the native peptide is believed to be altered upon introduction of PEG moiety, ultimately, affecting the activity of the conjugate. Moreover, owing to its steric interference, the PEG moiety may intemperately alter the key interactions responsible for primary activities of the protein/peptides^34, 66,67^. Another report on PEGylated HIV-1 derived fusion inhibitory peptides demonstrated that judicious selection of the site of PEG conjugation generates PEGylated peptides with only slightly reduced fusion inhibitory efficacies in contrast to the un-conjugated wild-type peptide, but with significantly improved proteolytic stabilities^68^. Thus, it may be permissible to conclude that the site and nature of PEG-conjugation has an impact on the properties of the resulting conjugates. Furthermore, in comparison to these previously reported studies, the decrease in activity of cryptdin-2 upon PEGylation was found to be very less (i.e. only twofold) in our study, which adds to its advantage.

Mammalian cell membranes are enriched with zwitterionic phospholipids displaying a net neutral charge on the surface, however, due to asymmetric distribution of phospholipids, hydrophobic interactions are sometimes found to overpower the comparatively weak electrostatic interactions between the host membrane and the peptide, thereby resulting in cytotoxicity^69^. A therapeutic intervention is considered to be reasonably efficient, if it is devoid of such toxicity and simultaneously has enhanced serum stability. It is worth noting that the cytotoxicity of the PEG-conjugated peptide was reduced, thereby, indicating that PEGylation had not evidently hampered the cell viability even at increased MIC, in contrast to the un-conjugated peptide forms which were detectably toxic to mouse macrophages at their respective MIC(s). These observations could be explained by possible steric interference caused by covalently attached PEG moiety, making it unable to permeate the extracellular matrix efficiently. Interestingly, PEG-modified peptide was observed to retain 70% of its activity up to 6 h in contrast to wild type recombinant peptide which retained its potency for 3 h only. The possible explanation for prolonged serum stability of PEGylated peptide might be the steric shielding effect offered by the PEG moiety, which is a well known phenomenon associated with these polymeric groups. The freely movable PEG moiety decorated at the peptide surface may restrict the interactions of modified peptide with proteolytic components of serum^70^. Therefore, it can be inferred that PEGylation certainly helps in increasing the serum stability, which in fact is desired for translation of these molecules into its clinical applications.

Thereafter, the developed designer peptide was evaluated for its antagonistic activity against *S. aureus* in combination with the routinely used antimicrobial agents such as gentamicin^71^. In the present scenario of emerging antimicrobial resistance, strains developing resistance against methicillin and vancomycin are on the rise and have rendered the current antimicrobials ineffective^72^. Interestingly, non-beta lactam drugs such as gentamicin, co-trimoxazole and clindamycin, against which resistant strains had been reported in the past^73–76^, are still being used to treat *Staphylococcus* mediated infections owing to their ability to be effective when used in combination. Resurgence in sensitivity of such antibiotics requires special attention for strengthening the available reservoir of antimicrobial compounds. Therefore, it is the right time to make efforts to retain the re-gained efficacy of gentamicin against methicillin-resistant *Staphylococcus aureus* (MRSA). In this regard, the combination of gentamicin with cryptdin-2, as evidenced by FIC index, can serve as a potential treatment option. Pore-forming ability of these peptides^77^ simultaneously promoting influx of gentamicin in the bacterial cells, eventually inhibiting protein synthesis, would have resulted in effective killing, even at lower concentrations. This decrease in the effective concentration of both the agents further gives credence to the ability of these peptides in not only reducing host cell toxicity but also preventing the occurrence of resistance due to their multipronged action. Similar studies conducted in the past have documented the efficacy of using AMPs such as cryptdin-2 and nisin with conventional antibiotics in vitro as well as in vivo^28–30,37^. Additionally, no change in the synergistic profile of the PEGylated peptide, in comparison to the wild-type cryptdin-2, when used in conjunction with gentamicin against *S. aureus*, further strengthens the hypothesis of using the two agents together. The present study is indicative of rational use of previously efficacious antibiotics in combination with other molecules in order to maintain the sensitivity and to combat the emerging resistance in pathogens like *S. aureus*.

## Conclusion

From the present study, it can be concluded that although PEGylation of cryptdin-2 resulted in slight increase in its MIC, but the overall potency of the peptide was enhanced as evidenced by: (a) increase in host cell viability; (b) better serum stability; and (c) equivalent efficacy when used in combination with conventional antibiotic. More importantly, the study demonstrates significant preliminary evidence as a proof of concept for developing peptide-based therapeutics for human use, particularly HD-5, which is the human counterpart of cryptdin-2. Currently, in this regard, the following strategies are being employed: (i) optimization studies regarding the size and site of PEG-conjugation in order to restore its original antimicrobial activity; (ii) evaluation of in vivo efficacy of PEGylated/un-PEGylated cryptdin-2, alone and in conjunction with gentamicin using *S. aureus* infected wound model as well as with other antibiotics in systemic infections caused by the pathogen. The combination of peptide with gentamicin might help in rejuvenating the use of conventional anti-Staphylococcal antibiotics in the treatment of multi-drug resistant *S. aureus* infections.

## Supplementary information

Supplementary information

## Data Availability

All data generated or analyzed during this study are included in this manuscript (and its Supplementary Information files).
