# Supplementary material for: Recombinant expression, purification and PEGylation of Paneth cell peptide (cryptdin-2) with value added attributes against Staphylococcus aureus
Source: Sci Rep. 2020 Jul 22;10:12164. doi: 10.1038/s41598-020-69039-2 (PMC7376037; doi:10.1038/s41598-020-69039-2)
Supplement: Supplementary file 1 — Supplementary information [file 41598_2020_69039_MOESM1_ESM.pdf]

Recombinant expression, purification and PEGylation of Paneth cell peptide (cryptdin-2) with value added attributes against *Staphylococcus aureus*

Navneet Kaur[1, 2], Rahul Dilawari[2], Amrita Kaur[1], Girish Sahni[2], Praveen Rishi[1*]

1. Department of Microbiology, Panjab University, Chandigarh, India
2. CSIR- Institute of Microbial Technology, Sector-39A, Chandigarh, India

*Corresponding Author

Email: rishiparveen@pu.ac.in

Supplementary data

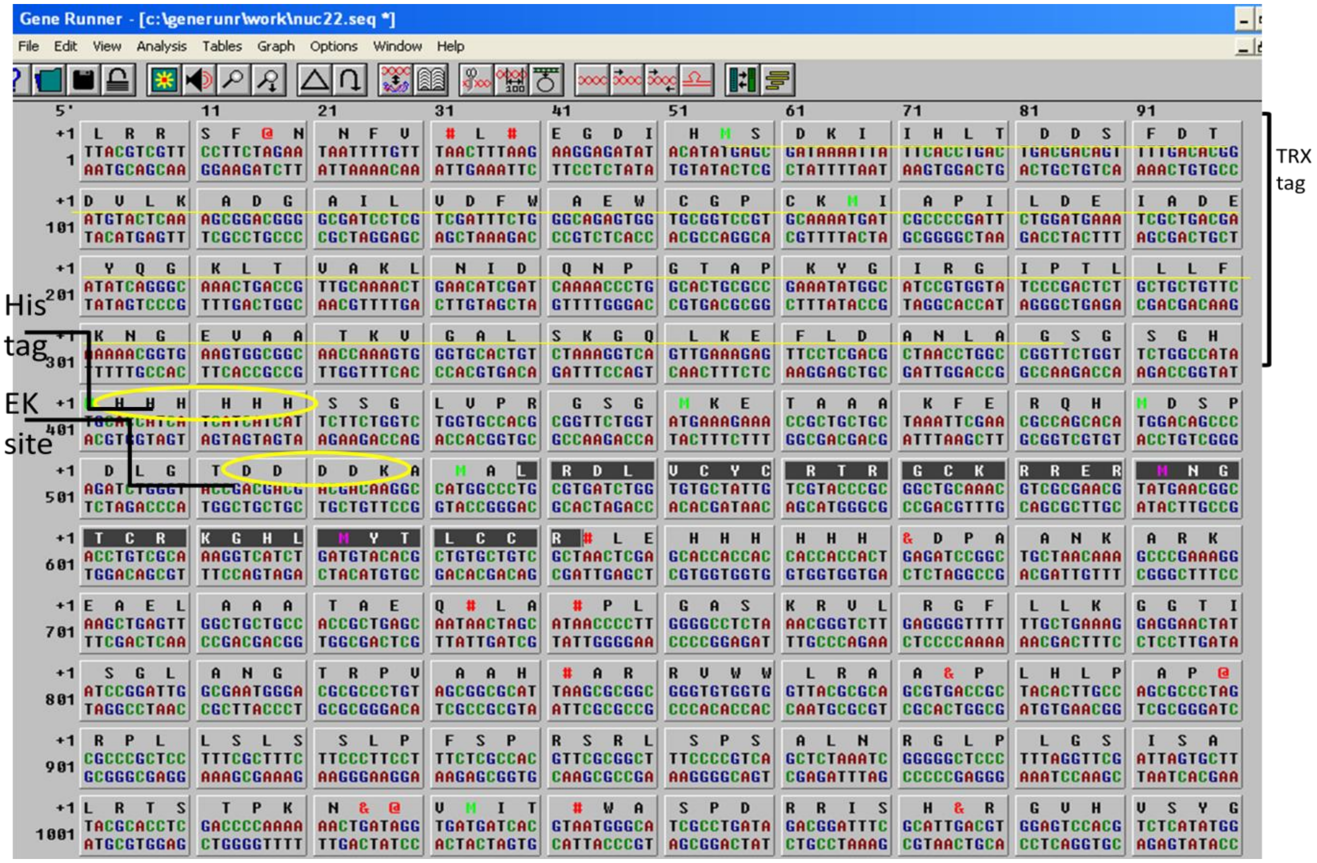

S1. DNA sequence (translated to amino acid sequence) of the prepared plasmid construct showing the position of N-terminal Thioredoxin tag, His6-tag and Enterokinase cleavage site (as confirmed by DNA sequencing)

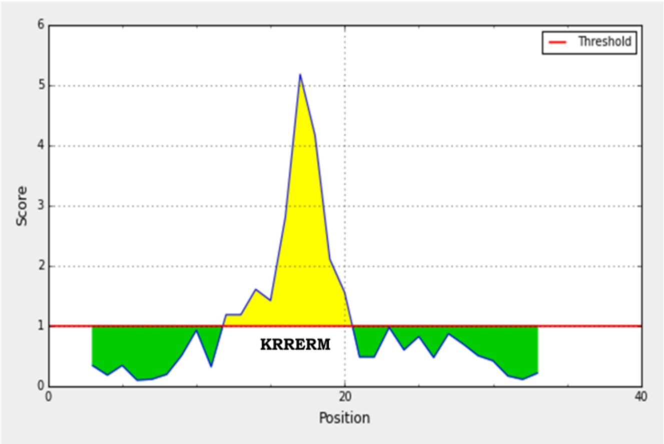

S2. IEDB Emini Surface Accessibility Prediction (KRERM-5.1 score) (http://tools.iedb.org/bcell/)

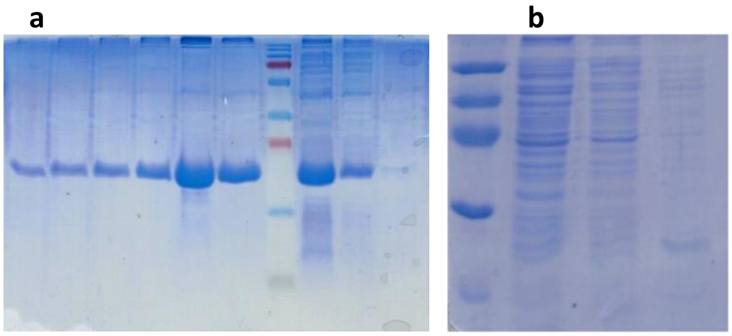

S3. SDS-PAGE profile of purification samples of fusion protein (Trx-His-Crp-2) showing **a.** Purified fractions and preload sample; **b.** load flow through and wash flow through samples

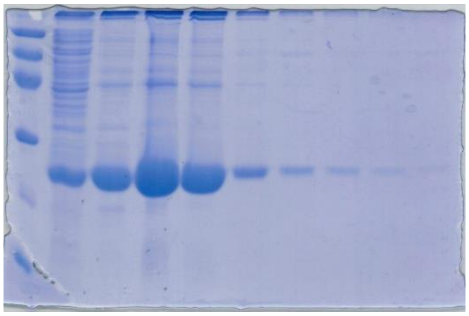

S4. Un-cropped SDS-PAGE profile corresponding to gel picture (B) of Fig. 1. in the manuscript)

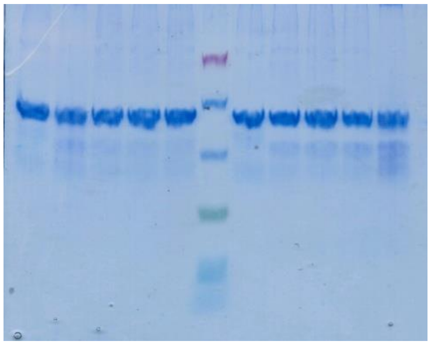

S5. Un-cropped SDS-PAGE profile of purified fractions of mutant fusion protein (corresponding to gel picture (C) of Fig. 1. in the manuscript)

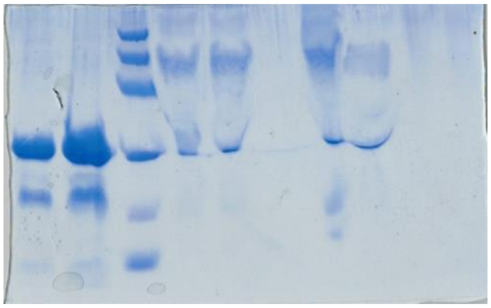

S6. Un-cropped SDS-PAGE corresponding to gel picture (A) of Fig 2. in the manuscript

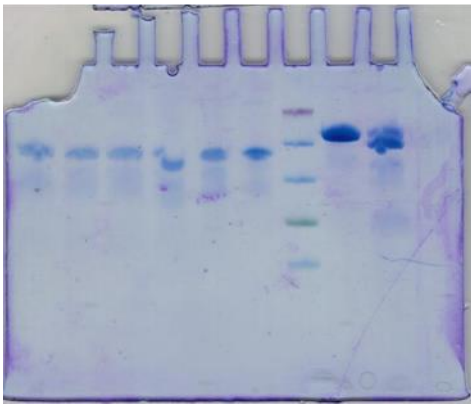

S7. Un-cropped SDS-PAGE corresponding to gel picture (B) of Fig 2. in the manuscript

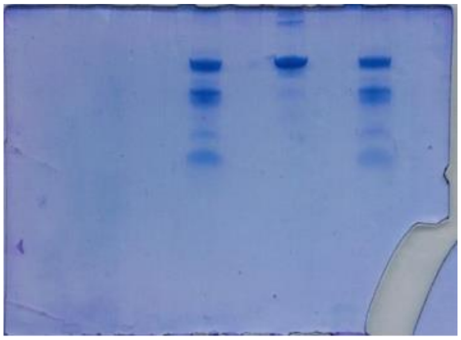

S8. Un-cropped SDS-PAGE corresponding to gel picture (B) of Fig 3. in the manuscript

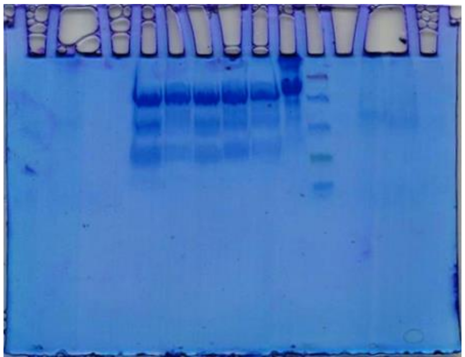

S9. Un-cropped SDS-PAGE corresponding to gel picture (D) of Fig 3. in the manuscript

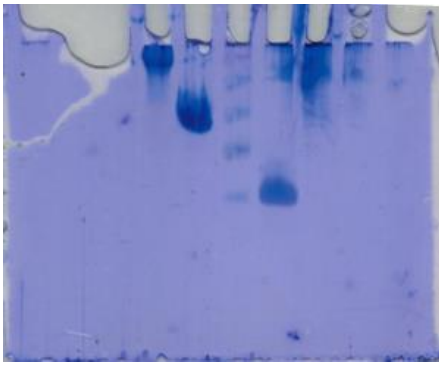

S10. Un-cropped SDS-PAGE corresponding to gel picture (B) of Fig 4. in the manuscript

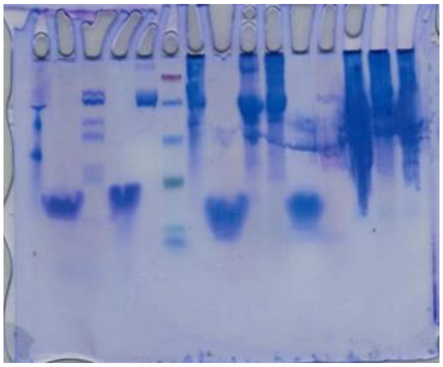

S11. Un-cropped SDS-PAGE corresponding to gel picture (C) of Fig 4. in the manuscript

S12. MICs of the peptides after incubation with serum

	MIC(s) (µg/ml)		
Time of incubation	cryptdin-2	mutant	PEGylated
0h	20	20	40
3h	40	40	40
6h	70	60	60

S13. The scheme of FIC checkerboard assay showing 64 combinations used to determine FIC.

	**Peptide (cryptdin/mutant/PEGylated)**							
**Gentamicin**	0.016MIC (P)<br>0.016MIC(G)	0.032MIC(P)<br>0.016MIC(G)	0.063 MIC(P)<br>0.016MIC(G)	0.125 MIC(P)<br>0.016MIC(G)	0.25 MIC(P)<br>0.016MIC(G)	0.5 MIC(P)<br>0.016MIC(G)	MIC(P)<br>0.016MIC(G)	2 MIC(P)<br>0.016MIC(G)
	0.016MIC(P)<br>0.032MIC(G)	0.032 MIC(P)<br>0.032MIC(G)	0.063 MIC(P)<br>0.032MIC(G)	0.125 MIC(P)<br>0.032MIC(G)	0.25 MIC(P)<br>0.032MIC(G)	0.5 MIC(P)<br>0.032MIC(G)	MIC(P)<br>0.032MIC(G)	2 MIC(P)<br>0.032MIC(G)
	0.016MIC(P)<br>0.063MIC(G)	0.032 MIC(P)<br>0.063MIC(G)	0.063 MIC(P)<br>0.063MIC(G)	0.125 MIC(P)<br>0.063MIC(G)	0.25 MIC(P)<br>0.063MIC(G)	0.5 MIC(P)<br>0.063MIC(G)	MIC(P)<br>0.063MIC(G)	2 MIC(P)<br>0.063MIC(G)
	0.016MIC(P)<br>0.125MIC(G)	0.032 MIC(P)<br>0.125MIC(G)	0.063 MIC(P)<br>0.125MIC(G)	0.125 MIC(P)<br>0.125MIC(G)	0.25 MIC(P)<br>0.125MIC(G)	0.5 MIC(P)<br>0.125MIC(G)	MIC(P)<br>0.125MIC(G)	2 MIC(P)<br>0.125MIC(G)

	0.016MIC(P)<br><br>0.25MIC(G)	0.032 MIC(P)<br><br>0.25MIC(G)	0.063 MIC(P)<br><br>0.25MIC(G)	0.125 MIC(P)<br><br>0.25MIC(G)	0.25 MIC(P)<br><br>0.25MIC(G)	0.5 MIC(P)<br><br>0.25MIC(G)	MIC(P)<br><br>0.25MIC(G)	2 MIC(P)<br><br>0.25MIC(G)
	0.016MIC(P)<br><br>0.5MIC(G)	0.032 MIC(P)<br><br>0.5MIC(G)	0.063 MIC(P)<br><br>0.5MIC(G)	0.125 MIC(P)<br><br>0.5MIC(G)	0.25 MIC(P)<br><br>0.5MIC(G)	0.5 MIC(P)<br><br>0.5MIC(G)	MIC(P)<br><br>0.5MIC(G)	2 MIC(P)<br><br>0.5MIC(G)
	0.016MIC(P)<br><br>MIC(G)	0.032 MIC(P)<br><br>MIC(G)	0.063 MIC(P)<br><br>MIC(G)	0.125 MIC(P)<br><br>MIC(G)	0.25 MIC(P)<br><br>MIC(G)	0.5 MIC(P)<br><br>MIC(G)	MIC(P)<br><br>MIC(G)	2 MIC(P)<br><br>MIC(G)
	0.016MIC(P)<br><br>2 MIC(G)	0.032 MIC(P)<br><br>2 MIC(G)	0.063 MIC(P)<br><br>2 MIC(G)	0.125 MIC(P)<br><br>2 MIC(G)	0.25 MIC(P)<br><br>2 MIC(G)	0.5 MIC(P)<br><br>2 MIC(G)	MIC(P)<br><br>2 MIC(G)	2 MIC(P)<br><br>2 MIC(G)

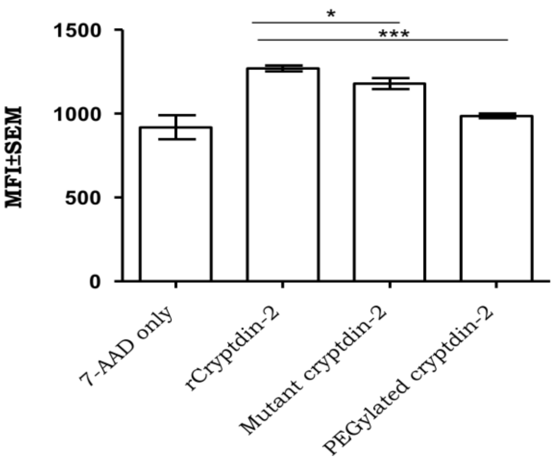

S14. Flow cytometry using 7AAD staining (After staining the bacterial cells treated with peptide variants, the fraction of dead population is maximum in wild type peptide. The dead bacteria fraction was lesser in mutant as well as pegylated peptide treated bacteria which is shown here using Flow cytometry using 7AAD staining and was statistically significant).
